# How to report perinatal and paediatric postmortem CT

**DOI:** 10.1186/s13244-024-01698-5

**Published:** 2024-05-31

**Authors:** Susan C. Shelmerdine, Owen J. Arthurs

**Affiliations:** 1 Great Ormond Street Hospital for Children NHS Foundation Trust, London, WC1N 3JH UK; 2 UCL Great Ormond Street Institute of Child Health, London, UK; 3 Great Ormond Street Hospital NIHR Biomedical Research Centre, London, UK

**Keywords:** Radiology, Autopsy, Paediatric, Postmortem, CT

## Abstract

**Abstract:**

Postmortem CT (PMCT) has become increasingly accepted alongside skeletal surveys as a critical part of investigation in childhood deaths, either as part of a suite of non-invasive investigations through parental choice, or comprehensive evaluation in a forensic setting. Whilst CT image acquisition and protocols have been published and are relatively standardised, CT imaging reporting remains highly variable, largely dependent upon reporter experience and expertise. The main “risk” in PMCT is the over-interpretation of normal physiological changes on imaging as pathological, potentially leading to misdiagnosis or overdiagnosis of the disease. In this article, we present a pragmatic standardised reporting framework, developed over a decade of PMCT reporting in children in our institution, with examples of positive and negative findings, so that it may aid in the interpretation of PMCT images with those less experienced in paediatric findings and postmortem imaging.

**Critical relevance statement:**

Standardised reporting using a common framework with a sound understanding of normal postmortem changes that occur in children are crucial in avoiding common reporting errors at postmortem CT.

**Key Points:**

- Familiarity with postmortem imaging is required for useful image reporting, and reporting standards vary.
- Understanding normal postmortem change from significant abnormalities requires training and experience.
- Following a template may remind reporters what to include and help improve performance.

**Graphical Abstract:**

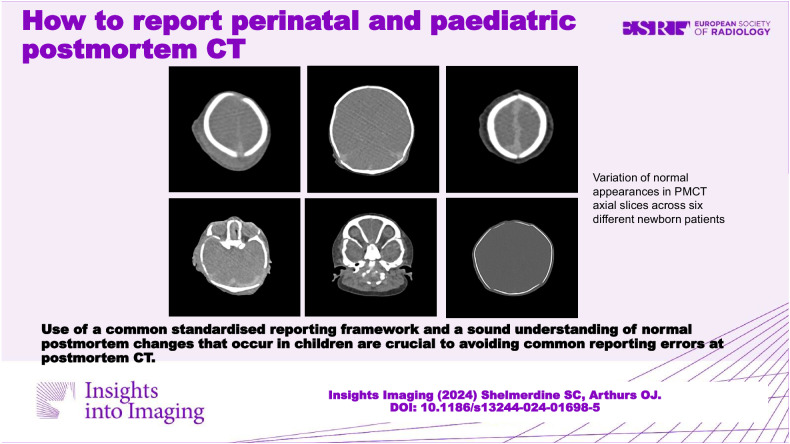

## Introduction

Cross-sectional postmortem imaging is becoming increasingly accepted alongside skeletal surveys as a critical part of the investigation into childhood deaths. Broadly, postmortem imaging is used as a non-invasive investigation in one of three ways: through parental choice (if perinatal), for coronial or medical examiner purposes (where death is non-suspicious but unexplained), or as part of a comprehensive evaluation in a forensic setting (if circumstances are potentially suspicious).

Whilst a variety of imaging modalities may be used (e.g., ultrasound [1], MRI [2], CT [3]), the use of CT is more widely adopted (estimated 51% of European centres in one survey) [4] and can be used to give a diagnosis, which may avoid invasive postmortem investigations [3, 5], provide valuable information regarding postmortem status and evaluate for subtle fractures, which may be highly relevant in a forensic setting.

It is important to acknowledge that postmortem CT (PMCT) will not provide a cause of death in all cases. In one study of 136 children, 56.6% (77/136) were found to have the cause of death at autopsy, with 71.4% (55/77) of these being identified at PMCT [3]. Understanding normal “physiological” postmortem change is critical to correct interpretation, and this experience forms the background upon which abnormal findings can be contextualised [6]. How to convey these findings to our forensic or pathology-referring colleagues requires skilled and clear communication, and just as across many other disciplines in radiology, imaging findings need to be appropriately contextualised against the clinical background to be most useful. Furthermore, it is important to bear in mind that any report could be read by the child’s family, and therefore considerate and respectful language should be used.

At present there is a lack of standardisation in reporting templates, which can lead to variability in practice, quality and comprehensiveness of PMCT reporting and also possible confusion regarding the usefulness of the imaging procedure. This paper tries to overcome some of these issues with the aim to reduce reporting uncertainty among practitioners. We summarise one such reporting strategy as a template checklist that can be adapted for use in clinical practice. We also explain the rationale behind some of the reporting terminology used to alert the reader in differentiating normal physiological postmortem change, postmortem artefact, or unexpected findings to ensure that potential pathological abnormalities across all systems have been thoroughly evaluated.

## How to refer

A minimum dataset on what information is required prior to performing in a PMCT child is needed and currently undergoing evaluation by the European Society for Paediatric Radiology (ESPR) postmortem task force. Generally, the age of the child, gender, antemortem clinical history and circumstances around the death should be made available to the reporter, in order to interpret certain findings correctly. As some findings will be iatrogenic during or after vigorous resuscitation, the extent of perimortem resuscitation should be well documented.

According to the latest guidelines from the Royal College of Pathologists on the investigation into sudden unexpected deaths in infancy and childhood [5], postmortem imaging investigations should never be undertaken without an expert external examination of the body having first been performed by an appropriately trained and experienced individual. The findings from this external examination may, therefore, also be helpful in informing potential sites of injury or helping to address areas of concern raised by the pathology/ mortuary team [7]. PMCT acquisition does not depend on the clinical history, but whether the body bag can be opened or must remain closed can affect patient positioning, and the decomposition state can be useful in interpreting imaging features subsequently.

## How to perform/acquire CT

Whole-body PMCT should be performed according to international guidelines. These have been published by EPSR (jointly with the International Society of Forensic Radiology and Imaging (ISFRI) [8]) and the Society of Pediatric Radiology (SPR) [9]. Whilst there are reports that have demonstrated some utility in ventilated PMCT [10] or the addition of intravascular contrast (e.g., PMCTA) [11, 12], neither is routinely practiced in children.

**Fig. 1:**
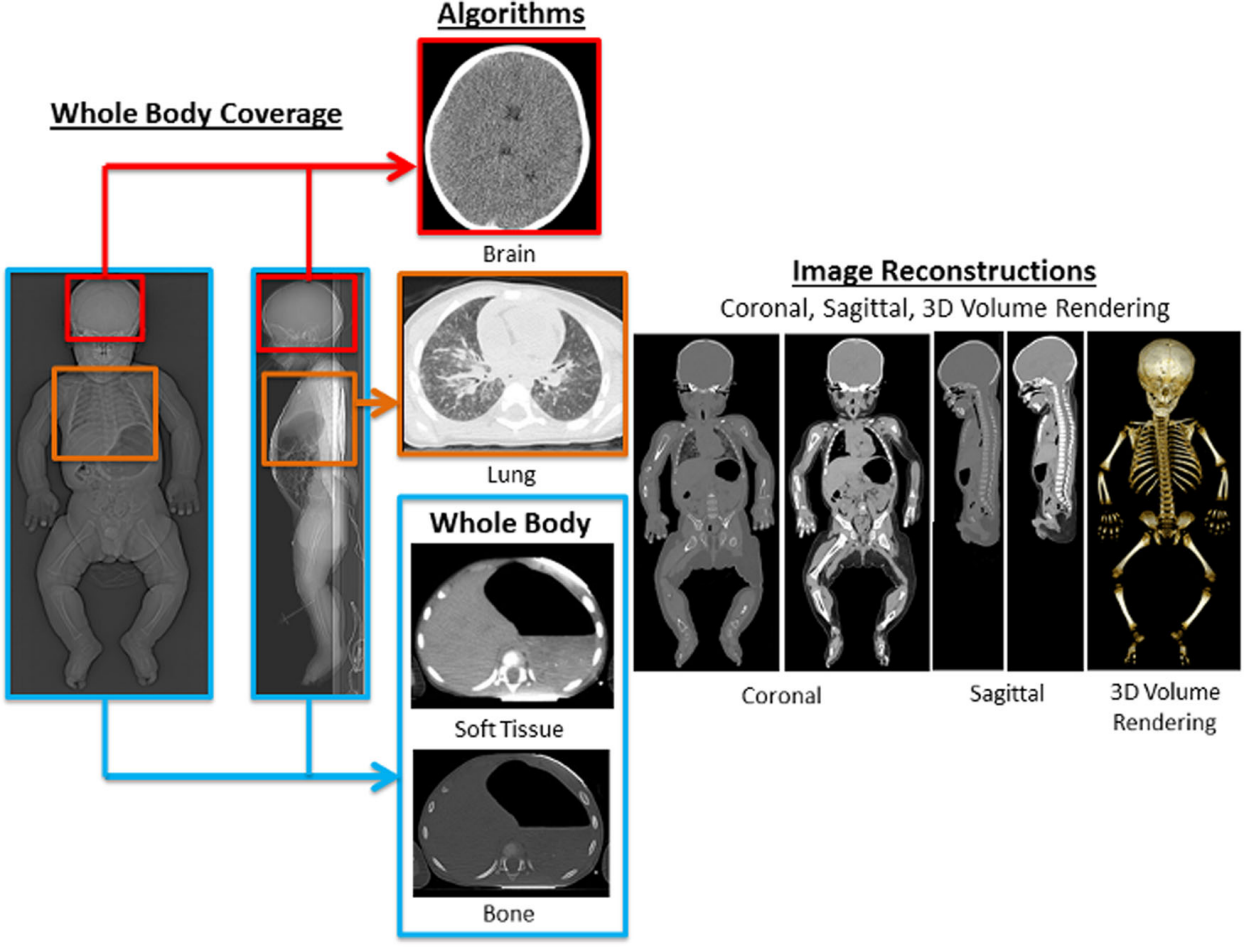
ESPR and International Society for Forensic Radiology and Imaging (ISFRI) recommendations for postmortem CT imaging in children. Whole-body coverage is recommended with region-specific brain (red box), lung (purple box) algorithms with also soft tissue and bone algorithms for the whole body (blue box). Different multiplanar reconstructions and volume rendering are also recommended. Reproduced without any adaptations from Shelmerdine S et al Pediatr Radiol **49**, 694–701 (2019) (http://creativecommons.org/licenses/by/4.0/)

The whole body should be imaged from the cranial vertex to the toes and can be performed in a single rotation then reconstructed separately into brain, body, and lower limb imaging, using appropriate body part-specific kernels and reviewed using the appropriate window settings (Fig. 1). Variation in the acquisition is unlikely to contribute to significant variation in interpretation, provided high-quality imaging is acquired at high resolution using sufficient radiation dose. There is little to be gained from not imaging the whole body, given that radiation dose and time of acquisition are not limiting factors in the postmortem setting. We advise against so-called “low dose” protocols, which reduce image quality; dose-optimised CT protocols for children should be used, which already have with sufficient dose as to allow for the identification of subtle findings which can be critical in suspected physical abuse.

3D reformatting can be performed according to personal preference but can be very useful for reviewing for fractures of the skull and ribcage, which are recognised to be easier to miss on axial imaging [13, 14].

## Who should report?

Given the workforce shortages across both radiology [15, 16] and pathology [17] medical specialties, adult postmortem imaging guidance has suggested that the imaging reporter need not necessarily be a radiologist (i.e., a “practitioner with competences in cross-sectional imaging working under the supervision of a radiologist”) [18]. This implies that a reporting pathologist or radiographer may be permissible, with a final report authorised by a radiologist. Given the complexity and major differences in pathology and causes of death in paediatric diseases, as well as the potential for forensic implications, in the UK, the Royal College of Pathologists guidelines mandate that a paediatric radiologist should report all paediatric postmortem imaging [5].

Currently, there is no requirement for paediatric postmortem imaging to be double reported (unlike in the setting for skeletal surveys for suspected physical abuse [19]) although this approach will clearly be helpful and practical for those less experienced or working in a centre where several radiologists are keen to develop and maintain postmortem reporting skills given the relative rarity of such examinations (compared to ‘live’ cases).

## What does the referrer want to know?

As already stated, the aim of a PMCT report is to help understand the cause of death or, where this is not possible, to provide additional information (i.e., important positive and negative findings) that may help inform further postmortem investigations where required.

To this end, it is important to understand the key questions that the referrer will be most interested in [5], for example:

- What are the key abnormalities present?
- Can these abnormalities be explained by a natural disease process or event; is there evidence of underlying disease?
- Is there evidence of non-accidental injury (suspected physical abuse)?
- Do the abnormalities remain unexplained, and would tissue sampling help?

Bearing these questions in mind whilst evaluating the imaging will enable a more clinically useful report to be issued. Teamwork is an important part of PMCT reporting, and if there is any uncertainty regarding key findings or unusual appearances, then these should be raised with the referrer.

## How to report

### General observations

First, make some general observations about the quality of the imaging or overall appearances of the patient. Comment on whether they are appropriately grown for age or gestation, using appropriate fetal reference tables where necessary.

Any iatrogenic support apparatus is also useful to identify and mention, including their position (endotracheal tubes, nasogastric tubes, internal jugular lines, tunnelled lines, vagal nerve stimulators, chest drains, etc.), although their position on the PMCT may not necessarily reflect their position antemortem so conclusions around whether these may have contributed to death should be drawn with caution. Nonetheless, an appreciation for the presence of support apparatus may infer the circumstances of the death or provide comment on the attempted perimortem resuscitation, such as misplaced tubes or lines.

Comment on whether any dysmorphic appearances or skeletal features are present and could raise the possibility of an underlying syndrome or skeletal dysplasia. Comment on muscle to fat ratio where possible, which may be important in medicolegal cases where neglect or starvation has been a feature.

The state of decomposition, subcutaneous oedema or widespread disseminated gas should be reported at the beginning. Subcutaneous oedema occurs during multi-organ failure and sepsis from any cause and may simply reflect the state of unwellness of the child but can be extensive and should not be interpreted as normal postmortem change. Typically, very little diffuse subcutaneous oedema is seen on most childhood PMCT outside of the setting of sepsis.

**Fig. 2:**
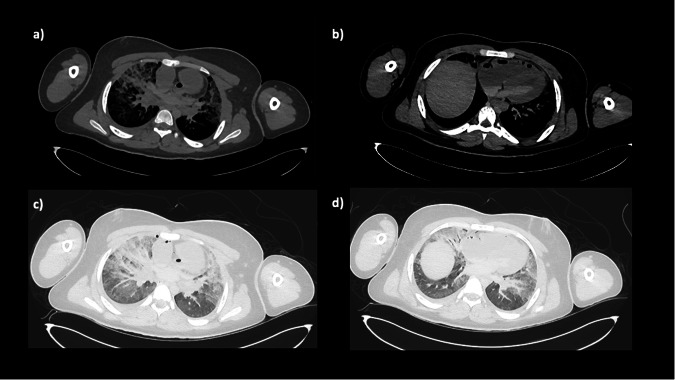
Axial PMCT of the chest in a 15-year-old boy in soft tissue (**a**, **b**) and lung (**c**, **d**) windows. Axial imaging at the level of the great vessels (**a**, **c**) shows nondependent gas in the heart, and density within the pulmonary trunk, which is normal venous stasis but could easily be mis-interpreted as pulmonary embolus. Axial imaging at the level of the heart (**b**, **d**) shows that good contrast is possible in older children without exogenous contrast material, and there is normal layering of the blood within the cardiac chambers due to sedimentation. On lung windows (**c**, **d**), the lungs show non-specific septal oedema with patchy opacification throughout, which is normal postmortem atelectasis

**Fig. 3:**
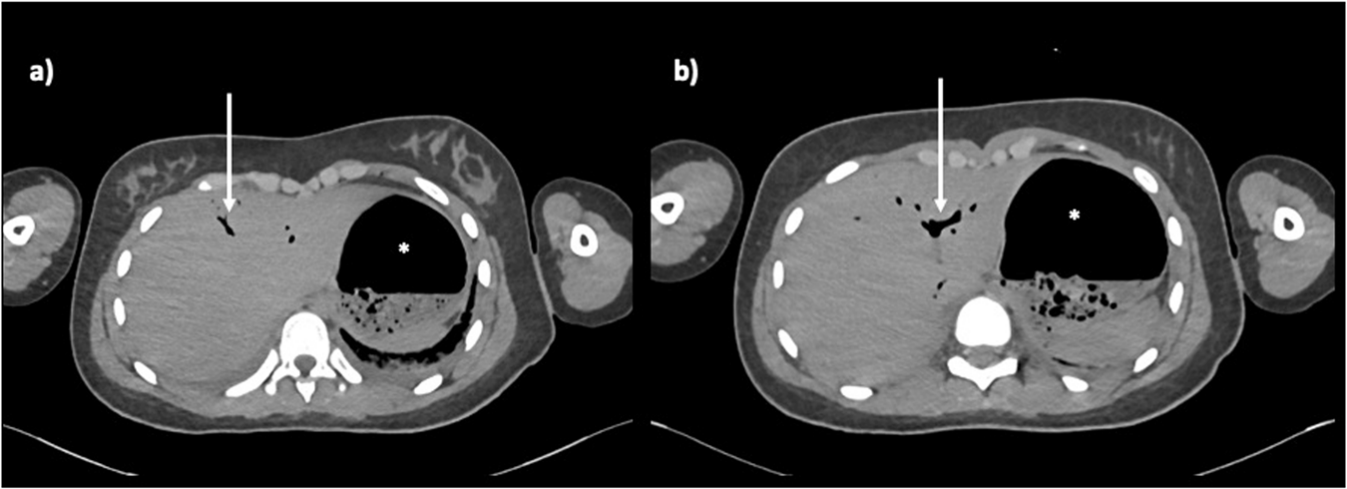
Axial PMCT imaging through the liver in a 13-year-old female at the level of the hepatic dome (**a**) and porta hepatis (**b**) both show normal gaseous gastric distension (asterisks) and non-dependent gas in the liver (arrows), which are normal postmortem findings. Note normal basal atelectasis in the left lung base

Typically, gas bubbles out of the blood and settles causing nondependent bubbles in the great vessels, hepatic veins, and particularly the right side of the heart-- these are normal postmortem changes that can be seen on either CT or X-ray (Figs. 2 and 3).

**Fig. 4:**
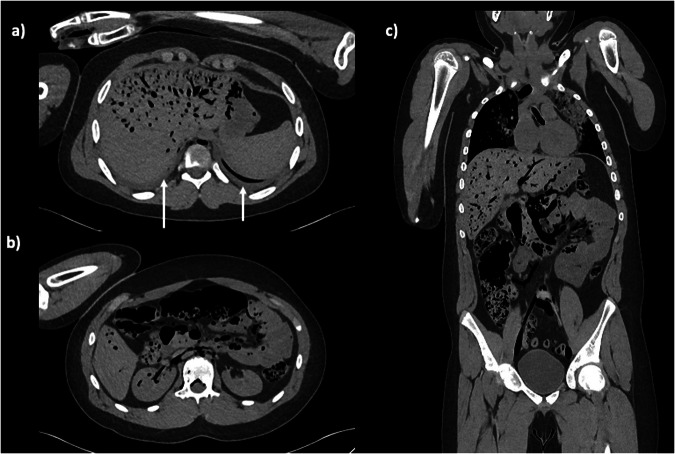
Axial PMCT images through the abdomen (**a**, **b**) and coronal images of the thorax and abdomen (**c**) demonstrating widespread disseminated gas, far more than in Fig. 3. The extensive nondependent gas is seen within the portal vein and branches, several mesenteric vessels and renal veins. This suggests gastrointestinal infection, likely anaerobic organisms. Note the small, normal postmortem pleural effusions (**a**, arrows)

Disseminated intravascular gas can be seen throughout the body as a true abnormality, for example, in fetal or neonatal cases of ascending infection from the placenta or severe anaerobic sepsis. Otherwise, disseminated intravascular gas can be seen following resuscitation, whereby we hypothesise that vigorous chest compressions may move some intra-cardiac gas around the body [20]. Specific location of pockets of intravascular gas can be useful to mention, for example widespread mesenteric and hepatic gas in suspected gastrointestinal infection (Fig. 4).

**Fig. 5:**
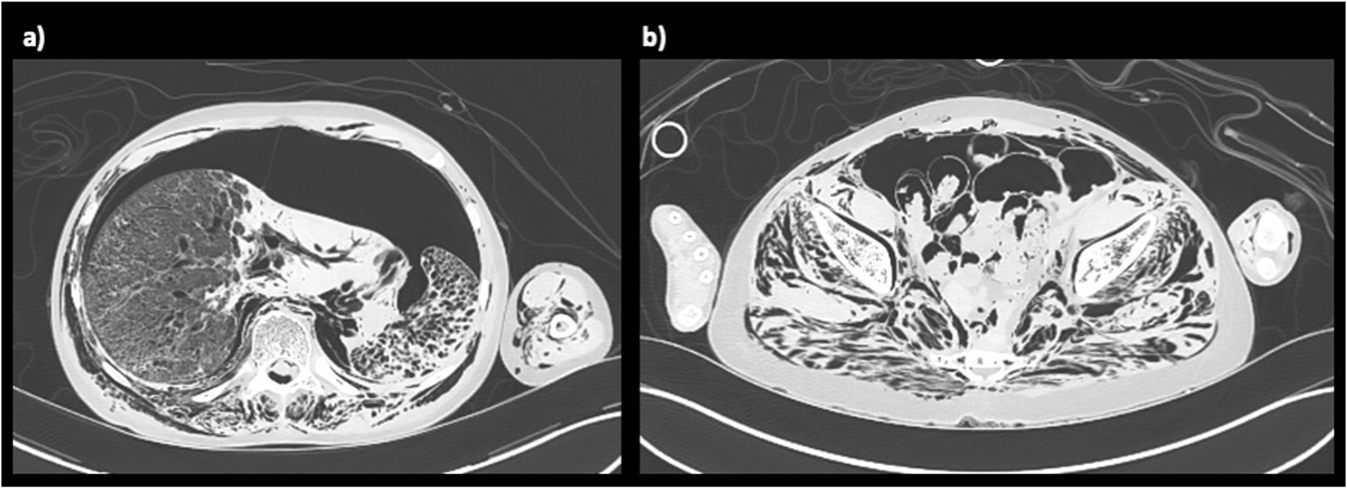
Axial PMCT images through the upper abdomen (**a**) and pelvis (**b**) of a 10-year-old girl whose body was undiscovered for several days during hot weather. There is disseminated gas throughout the liver, spleen, abdominal wall and particularly the lumbar and gluteal muscles in keeping with decomposition and bloating

Body decomposition can affect CT imaging in multiple ways, usually either gas changes to the subcutaneous tissues, bloating or insect infestation, which can give rise to unusual appearances (Fig. 5). Bloating is unusual in the absence of heat, e.g., if the body has been discovered during hot weather.

### Central nervous system

**Fig. 6:**
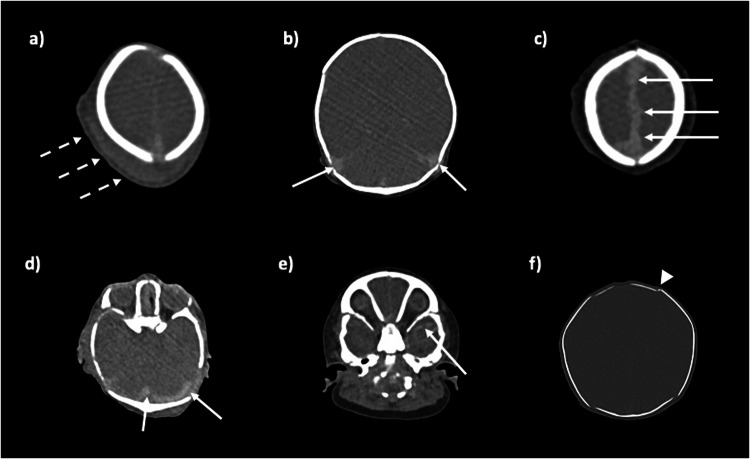
Variation in normal postmortem appearances of the brain at PMCT in several different newborns, axial section. Loss of grey-white matter differentiation and cerebral oedema are normal postmortem brain imaging features (**a**–**f**). Soft tissue scalp swelling is common after vaginal delivery (**a**, dashed arrows), and can be asymmetric and high density, in which case it may be a cephalhaematoma. High-density blood in the venous sinuses is normal postmortem venous stasis (**b**–**e**, solid arrows). The globes are collapsed and lens opacified (**d**), and there may be some overlapping of sutures, which should not be confused with a skull fracture (**f**, arrowhead)

It is important to recognise that death is a hypoxic process, and so the normal changes expected with brain hypoxia occur. Whether antemortem, perimortem, or postmortem, hypoxia results in the same appearances on CT: non-specific cerebral oedema, loss of grey-white matter differentiation, and then collapse of the globes and opacification of the lens (Fig. 6). Important negatives to mention are the absence of major abnormalities, mainly haemorrhage in the postmortem setting (ESM, Figure S1). Cerebral venous stasis, particularly at the vertex and along the falx, appears dense and can be easily mis-interpreted as subdural haemorrhage or sinus thrombosis; increased experience is useful to help differentiate between normal and abnormal appearances (Fig. 6).

Skull fractures should be evaluated using 3D reformats, and the presence or absence of accessory sutures and Wormian bones is helpful [21]. Check the skull base for fractures which extend across and may involve the sphenoids or sinuses (ESM, Figure S2). The spinal cord can be difficult to visualise on unenhanced CT, but any vertebral trauma can direct the reporter’s attention to potential spinal impingement as well as haemorrhage. Again, normal postmortem venous stasis, particularly around the foramen magnum, should not be mis-interpreted as haemorrhage.

### Neck

In the trauma setting, the main normal feature to evaluate is vertebral alignment and the presence/absence of soft tissue swelling. These are not specific features, and the absence of swelling does not exclude significant neck trauma.

There is often atlantoaxial rotatory subluxation at death due to ligamentous laxity, which makes this diagnosis in the postmortem setting particularly challenging. The body may have been positioned in a particular orientation for CT, which may also contribute to apparent atlanto-axial subluxation. These features make diagnosing true abnormalities challenging, for example, in teenage drowning where diving head first into a shallow swimming pool has resulted in C-spine trauma.

The thyroid and hyoid are usually easy to identify in normal children. The question of hyoid integrity in strangulation is one which occurs in adult trauma; the hyoid is relatively plastic in children and does not often fracture, even in cases of strangulation. Whilst the presence of hyoid fracture in older children implies neck trauma, the absence of evidence (intact hyoid) is not evidence of the absence of trauma in this context.

### Chest

**Fig. 7:**
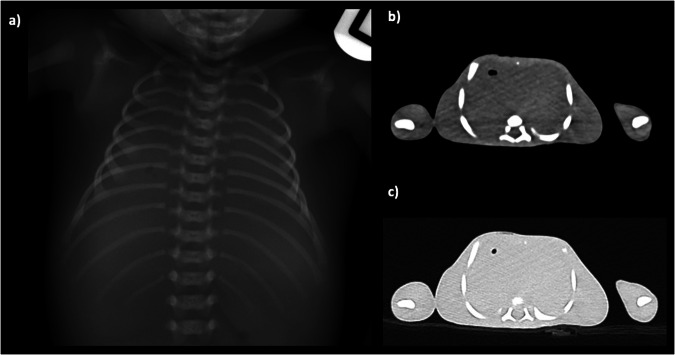
Postmortem chest radiograph (**a**) and axial PMCT images of the lungs in soft tissue (**b**) and lung windows (**c**) in a newborn term baby. There is minimal soft tissue contrast differentiation making cardiac and lung imaging nondiagnostic

There is a large spectrum in normal appearances of the lungs in the postmortem state, and this makes interpretation of the lungs on paediatric PMCT the most challenging area to report correctly. The lungs may be normally well aerated with only minor patches of postmortem collapse, which mimic non-specific ground glass opacification in atypical infection in life. They may be completed fluid filled and collapsed, with large simple pleural effusions, which can also represent normal (physiological) postmortem change (Fig. 7). The reasons behind this spectrum of appearances are poorly understood, and there is limited evidence that the appearances are related to underlying pathology or time since death: there is poor radiological-pathological correlation even amongst experienced postmortem radiologists in this regard [3]. The most *useful* and *accurate* way to report these changes is, therefore, factual, making minimal inference. For example, “bilateral patchy consolidation, which may be normal postmortem change” is more helpful to the pathologist than “bilateral patchy consolidation which may reflect infection”, although both may be equally correct. Even markedly asymmetric changes can be normal.

**Fig. 8:**
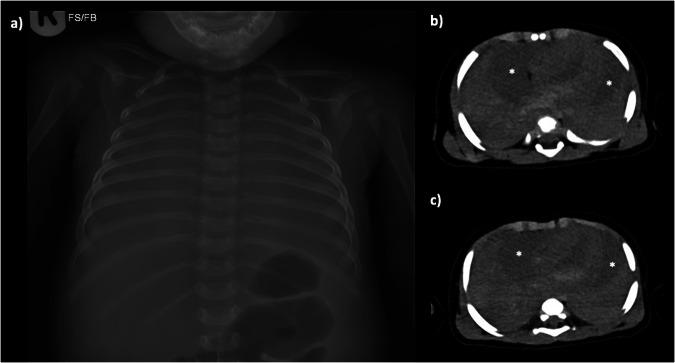
Postmortem chest radiograph (**a**) and axial PMCT images through the thorax (**b**, **c**) in a 5-month-old girl found unresponsive. The PMCT demonstrates a large pericardial effusion (asterisks) with cardiomegaly, not visible on chest radiography. In the absence of a history of peri-resuscitative or postmortem needle sampling of this fluid, it should be presumed to be an antemortem. The dense consolidated appearances of the lungs were considered normal postmortem change

However, whilst small simple pleural and pericardial effusions are normal in the postmortem setting, their volume is likely dependent on time since death, since the accumulation of fluid in the pleural space has been identified to occur on sequential imaging [22] (Fig. 8). Any blood or gas in the pleural spaces cannot be attributed to normal postmortem change, and so a chest wall penetrating wound or rib fracture should be sought. These may be iatrogenic, for example, bilateral chest drains placed during helicopter retrieval of a trauma victim to the hospital.

Trauma to the chest wall can result in rib fractures or subcutaneous findings, which are usually obvious in violent deaths involving shooting or stabbing. Here, volume rendering can be useful to identify superficial puncture wounds which may not be obvious on visual inspection, PCMT can help differentiate penetrating from non-penetrating trauma, as tracking of gas or blood in the soft tissues can indicate depth of penetration.

Rib fractures are one of the areas where PMCT is particularly sensitive in the childhood death arena. Rib fractures anywhere on the rib are easier to detect on CT than chest radiography [14], and rib fractures may be the only marker of child abuse [23]. Any rib fractures must be mentioned and categorised, together with the presence or absence of any healing response.

**Fig. 9:**
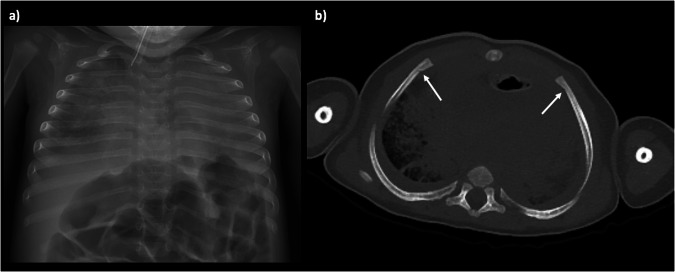
Postmortem chest radiograph (**a**) and axial PMCT image through the thorax (**b**) in the same infant demonstrating bilateral symmetrical greenstick rib fractures of the ribs on PMCT (arrows), around 2 cm posterior to the costochondral junction caused by resuscitation, which are difficult to see on plain radiographs, but in a typical expected location

Chest compressions during vigorous conventional cardiopulmonary resuscitation can cause anterior rib fractures, although the precise depth or force required to induce these in a fairly plastic ribcage remains unknown. Whilst there is limited evidence about the exact force or duration of cardiopulmonary resuscitation required to cause rib fractures, the incidence of resuscitation-related rib fractures on postmortem CT is significantly higher than on conventional radiographs as they are much easier to identify [14]. It is now widely accepted that multiple bilateral symmetrical anterior greenstick rib fractures of the 2–7th ribs inclusive, in a line around 2 cm posterior to the costochondral junction, can be caused by resuscitation [24] (Fig. 9). They are incomplete, greenstick fractures with buckling of the internal cortex leaving the outer cortex intact, as children’s ribs are plastic and resist significant deformation before buckling. Any fractures which are complete (nongreenstick) may be caused by direct impact trauma, and care should be taken before attributing these to resuscitation. A review of 3D reformats, particularly with angling of the chest wall to evaluate the symmetry of both clavicles or scapulae, is useful to evaluate for fractures to the shoulder girdle.

### Heart

Using unenhanced PMCT to comment on the heart can be challenging and must only be done within the limitations of what is possible. For example, in a small fetus with very little contrast to noise, the cardiac imaging may be nondiagnostic (Fig. 7). In neonate or older children, imaging of the heart may be used to include or exclude major congenital heart disease. Blood and gas pooling in the heart chambers can cause a natural contrast medium against the heart wall helping to identify potential abnormalities. Intact septum, ventricular orientation, wall and lumen thickness should all be mentioned. Calcification of the ductus arteriosus remnant is common [25], is usually an incidental finding, and does not need to be mentioned.

### Abdomen

**Fig. 10:**
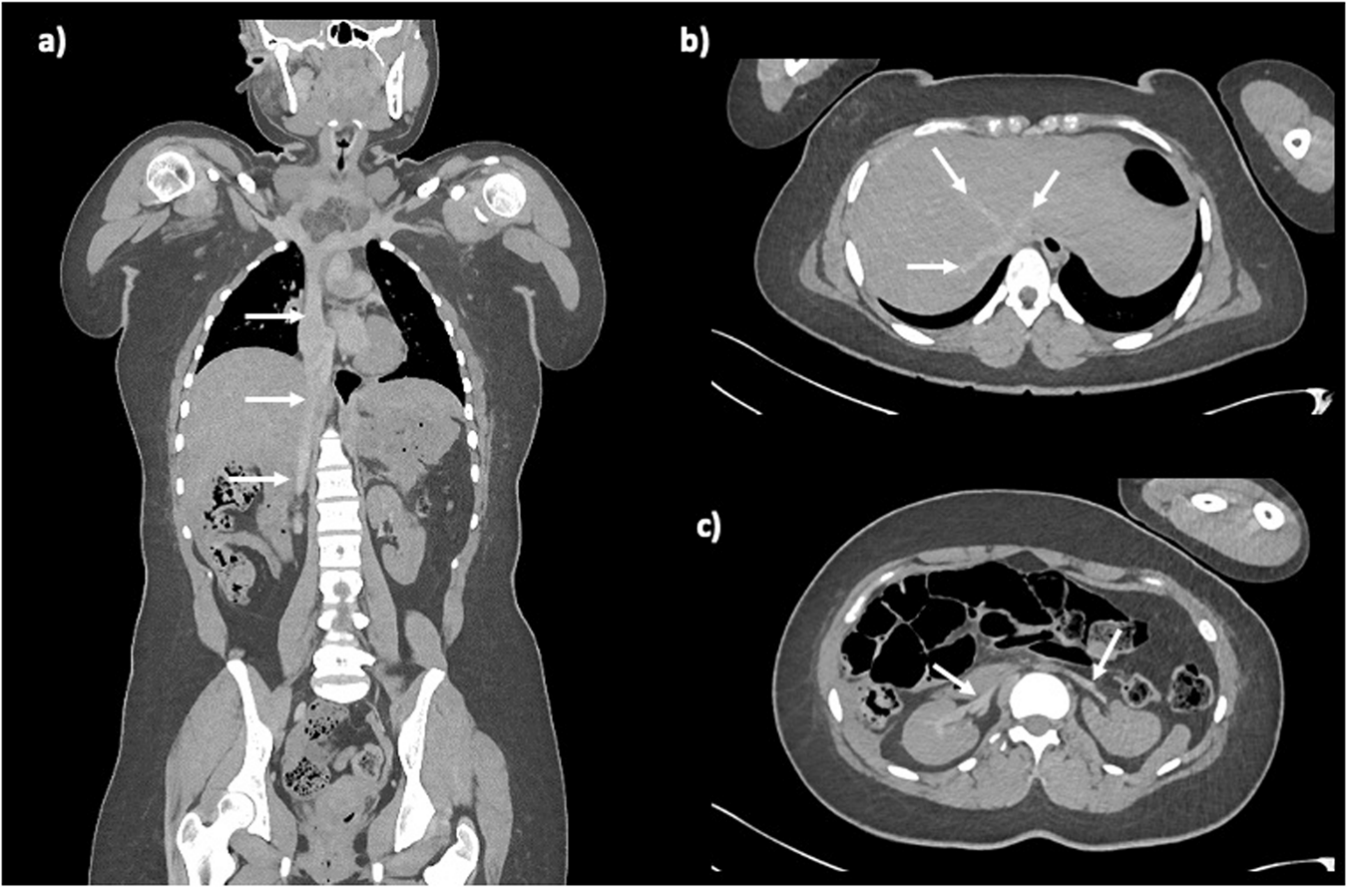
Normal PMCT abdominal findings. Coronal (**a**) and axial (**b**, **c**) PMCT images through a 16-year-old female showing hyperdense hepatic and renal veins and IVC (arrows). This is normal postmortem venous stasis and should not be interpreted as a thrombus

Unenhanced PMCT also has limitations regarding the diagnostic accuracy of the major abdominal organs, but their presence/absence, orientation and any congenital abnormalities are usually apparent with sufficient contrast to noise and intervening fat planes. Similarly, the diaphragm should be intact, and the great vessels should be easily identifiable from diaphragm down to the limbs, although the large veins often appear flattened or high density even in those with normal premortem circulation (Fig. 10).

**Fig. 11:**
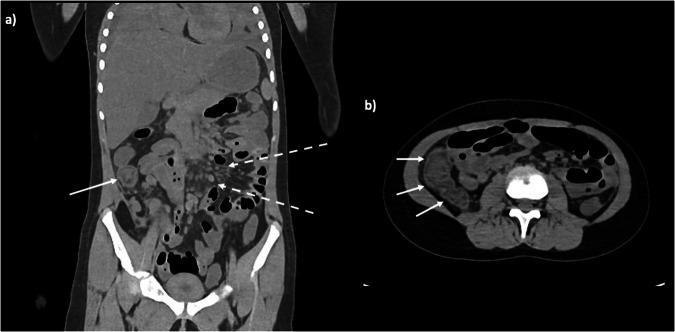
Coronal (**a**) and axial (**b**) PMCT images of an 8-year-old girl who died following a short diarrhoea and vomiting illness. The PMCT images showed bowel wall thickening in the right colon (solid arrows), and widespread mesenteric lymphadenopathy (dashed arrows)

Whilst unenhanced CT may not give the same versatility as live contrast-enhanced imaging, several abnormalities in the solid organs can be detectable. Low-density lacerations or high-density haemorrhage should be identifiable on the major organs, as should organomegaly or focal lesions, and overall density may be critical. One of the more straightforward abdominal imaging diagnoses is that of free gas, whether small locules or large volume, implying visceral perforation or infection. Bowel wall thickening can be identified in older children (Fig. 11), and calcification within the bowel lumen in the fetal setting is easy to detect, but rarely has any clinical significance [26] and reporting this as a “significant” finding on PMCT is usually not helpful. Like calcification of the ductus arteriosus, it should be considered an incidental finding.

A small volume of pelvic free fluid is almost universal in the postmortem setting, similar to pericardial or pleural effusions, but increased density proteinaceous ascites particularly in both paracolic gutters usually signifies haemorrhagic content, and a source of haemorrhage should be sought. Appropriate windowing of the imaging is useful to exclude hepatic, renal, or splenic lacerations – although these can be challenging to detect on unenhanced PMCT, and care should be taken when assuming an unenhanced pancreas is intact.

Dependent oedema within the buttocks or upper thighs can be normal postmortem changes, but focal soft tissue oedema, particularly around the groin or upper thighs, can usually be attributed to attempted vascular access.

### Musculoskeletal system

The presence or absence of vertebral, shoulder girdle, pelvic, and limb fractures or bone abnormalities should be described in the same way as a live child’s trauma scan, with particular reference to vertebral alignment and any associated soft tissue abnormality which may represent haematoma. PMCT can be useful to evaluate the extent of bone disease in known antemortem disorders, such as disseminated malignancy.

Pelvic fractures can be subtle, but PMCT is particularly sensitive to diagnosis, and often identifies more fractures than appreciated on radiographs [14], in contrast to limb fractures, where CT is probably not as sensitive as radiographs, particularly for subtle corner metaphyseal lesions/fractures (CMLs) [27, 28]. It is still reasonable to perform appendicular radiographic imaging even when high-quality whole-body CT imaging has been performed, as both are complementary. Similarly, hands and feet are not well evaluated using whole-body CT.

**Fig. 12:**
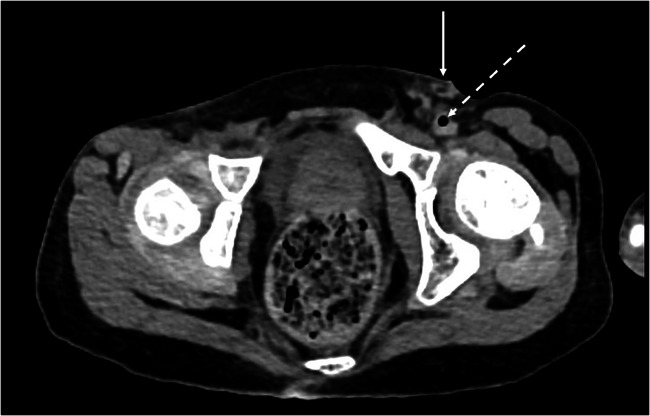
Axial PMCT image through the groin of a 16-year-old boy who was found unresponsive. Focal soft tissue oedema around the left groin (solid arrow) with a small locule of gas in the left external iliac vein (dashed arrow) is indicative of prior attempts at femoral intravenous cannulation by the ambulance team at the scene

If not already, mention all iatrogenic lines and tubes, as their position may be important. It is quite common for femoral venous lines to be attempted (and sometimes abandoned) at the scene of the emergency (Fig. 12) and also for intraosseous needles to be outside of the expected bone marrow position (often in the soft tissues, joints or traversed the bone in its entirety) [29, 30] (ESM, Fig. S3). This often results in multiple locules of gas within the vessels and soft tissues, fat stranding in the soft tissues, or asymmetrical soft tissue oedema of the limb.

### Review areas

To complete a PCMT report, a checklist of potential well-recognised “misses” at the end of the report can be useful to ensure that it is as comprehensive as possible. Lung reformats to evaluate for tiny pneumothorax or pneumoperitoneum can be useful, virtual rendering of the skin surface to identify any puncture marks, and zooming in on each joint to look for CMLs in multiple planes and 3D reformats can be particularly useful. Re-evaluating rib fractures in the context of any other abnormalities can be useful at this stage. Lines and tubes, if not already assessed, can be mentioned at the end of the report.

## Template reporting

To the unexperienced, template reporting may help the reporter to become more confident that they have covered the main detectable abnormalities on paediatric PMCT. We suggest that the use of a template can help ensure all areas are reviewed, but at the same time, multiple normal findings do not necessarily all need to be listed in a report unless these are pertinent to the referrer (i.e., to confirm the absence of injury where a finding has been raised on external examination). With a balance between pragmatism, time spent reporting and usefulness of the report to the referrer, we have found the following framework useful (Table 1), and our standard reporting template incorporates the clinical information of the patient and findings divided by subheadings of the different body systems (Table 2).

**Table 1:** Common findings on paediatric postmortem CT (PMCT)

Body part	Normal findings	Important negative findings	Common errors	Additional features as necessary
**Brain**	Hypoxic-ischaemic change (e.g. loss of grey-white matter differentiation) Cerebral oedema (i.e. globally reduced sulcation). Hyperdense venous sinuses due to stasis. Intracranial venous gas Sunken eyeballs and lens dislocation.	No haemorrhage. No ventricular dilatation. No space-occupying lesion.	Interpreting cerebral venous stasis as thrombus or haemorrhage. Trying to determine whether cause of death was due to hypoxic-ischaemic damage.	Intracranial gas may signify skull base fracture or open trauma elsewhere, e.g., neck or chest.
**Skull**	Intact (although sometimes overlapping sutures if newborn/fetal).	No fracture	Interpreting accessory sutures as fractures, the potential to miss fractures if 3D volume rendering is not used.	Mention Wormian bones if present.
**Spine**	Cord swelling Venous stasis.	Normal conus level. No paraspinal haemorrhage. No vertebral fractures (if trauma case or known bone fragility).	Interpreting cerebral venous stasis as thrombus particularly around foramen magnum.	Check occult spina bifida or segmentation anomalies.
**Neck**	Intravascular gas	No soft tissue swelling. Central venous lines positioned correctly.	Absence of hyoid bone fracture does not exclude neck trauma in infants and children	Disseminated intravascular gas may be secondary to resuscitation
**Chest (lungs)**	Collapsed lungs or patchy atelectasis Dependent lung changes Symmetrical pleural effusions. Fluid in the airways.	No focal consolidation or lung nodules. Chest drains and endotracheal tube appropriately sited.	Over-interpretation of patchy lung changes as pneumonia Over-interpretation of dependent venous stasis in main pulmonary artery as a pulmonary embolus.	Mention anterior rib fractures often following resuscitation. Look carefully for posterior rib fractures, which may signify inflicted injury.
**Chest (heart)**	Pericardial effusion. Ductus arteriosus calcification. Nondependent gas within the right heart. Collapsed aortic arch Dependent hyperdense blood sedimentation in all chambers and great vessels.	No septal defects. Normal orientation of main outflow tracts. Normal venous return.	Over-interpretation of septal defects (e.g. misinterpreting patent foramen ovale from atrial septal defect in younger children) Interpreting collapsed aortic arch as underlying coarctation Interpreting normal tissue laxity as pathological mediastinal shift. Over-interpretation of hyperdense vessels as thrombus within systemic veins (e.g. superior and inferior vena cava).	Comment on cardiac anatomy but acknowledge this can be challenging. Do not try to overinterpret findings in the heart.
**Abdomen**	Poor tissue contrast. Gaseous distension of stomach and bowel. Small-volume simple low-density free fluid. Mesenteric gas locules or portal venous gas.	Intact solid organs. No free air or dense proteinaceous ascites.	Over-interpretation of gastrointestinal tract dilatation as obstruction. Over-interpretation of mesenteric gas as bowel ischaemia. Missing solid organ laceration due to lack of tissue contrast on unenhanced imaging. Missing small locules of free air.	Comment on congenital anomalies.
**Pelvis**	Collapsed urinary bladder, Trace of low-density free fluid.	No dense free fluid	Missing subtle pelvic or sacral fractures Interpreting vascular channels as non-displaced fractures	Mention pelvic fractures, proteinaceous ascites.
**Lower limbs**	Age-dependent ossification. Physiological periosteal reaction. Intraosseous needles misplaced.	No fractures	Missing subtle corner metaphyseal lesions. Interpreting vascular channels as fractures. Insufficient acquisition to cover all 4 limbs.	Asymmetrical limb oedema (or localised soft tissue gas within the limb soft tissues) may indicate misplaced intraosseous needles.
**Upper limbs**	Age-dependent ossification. Physiological periosteal reaction.	No fractures	Missing subtle corner metaphyseal lesions. Interpreting vascular channels as fractures. Insufficient acquisition to cover all 4 limbs.	
**Skin**	Subcutaneous oedema Gas within subcutaneous tissues Surgical emphysema	No open trauma	Attributing localised gas to local trauma. Gas may have travelled from distant wound, particularly after vigorous resuscitation.	In some cases, it may be helpful to review volume rendering reconstructions to help assess external body features—e.g. ligation marks (suspected strangulation/hanging) or entry wounds (stabbings).

Note that not all the normal findings may be visible on all postmortem imaging

**Table 2:** Example of a standard reporting template with report text in a case without findings on PMCT

**Clinical indication**	9-month-old boy found unresponsive
**External examination findings**	No marks on the body of the note
**Technique**	Whole body unenhanced postmortem CT
**Coverage**	Vertex to toes
**Overall status**	Well-nourished child consistent with age/gestation. No subcutaneous oedema or gas, nor features of decomposition.
**Neurological system**	Normal postmortem intracranial and intraspinal appearances. No skull fracture or haemorrhage.
**Chest (cardiothoracic system)**	Normal collapsed lung appearances with postmortem pleural effusions. Conventional cardiac anatomy. No additional findings.
**Abdominal system**	Normal postmortem appearances to the solid organs. Small-volume proteinaceous ascites
**Musculoskeletal system**	No rib, vertebral or long bone fractures. Endotracheal tube, nasogastric tube and intraosseous needles appropriately sited.
**Conclusion**	Normal postmortem appearances.

## Conclusion

Overall, an appreciation of the clinical situation and some experience of expected postmortem appearances can help avoid both over- and under-interpretation at paediatric PMCT. Recognising that many apparently abnormal findings may represent normal postmortem change will give the reporter insight into how to report the most pertinent findings. Fundamentally, experience and feedback (either from autopsy findings or pathologist colleagues) across many PMCT examinations helps identify common postmortem changes so that when the unexpected is identified, a clear diagnosis can be made, and autopsy directed accordingly.

Starting this learning process can be daunting, especially if undertaken without local or even distant reporting support and mentorship. This may be one of the reasons why PMCT services are not as widespread and easily accessible as we would ideally like. Whilst there are some barriers to adoption (e.g., funding, training, and administrative processes [31–35]), several solutions are also possible and discussed in previous key publications issued by international societies, such as the ESPR and SPR taskforces [9, 31, 36–39].

One important factor in ensuring a quality and effective service is establishing clear routes of communication with the local pathology team and setting up regular multidisciplinary team discussions for feedback on the outcome of cases, as well as becoming members of shared professional groups (such as dedicated postmortem imaging taskforces) for case discussion, education and networking.

### Supplementary information

ELECTRONIC SUPPLEMENTARY MATERIAL

## Data Availability

Data sharing is not applicable to this article as no datasets were generated. The summary of findings from previously published articles are provided already in figures within this review.

## Abbreviations

CML: Corner metaphyseal lesion
CT: Computed tomography
ESPR: European society of paediatric radiology
ISFRI: International society of forensic radiology and imaging
PMCT: (Postmortem) computed tomography
SPR: Society of pediatric radiology
UK: United Kingdom
