# Supplementary material for: How to report perinatal and paediatric postmortem CT
Source: Insights Imaging. 2024 May 31;15:129. doi: 10.1186/s13244-024-01698-5 (PMC11139809; doi:10.1186/s13244-024-01698-5)

# How to report perinatal and paediatric postmortem CT
# ELECTRONIC SUPPLEMENTARY MATERIAL

**Figure S1.** Axial PMCT slices (a, b) and coronal midline slice (c ) through the brain of a premature 26 week gestation male born in poor condition show abnormal high grade intraventricular haemorrhage throughout the CSF spaces (b, c). Note loss of grey white matter differentiation throughout the brain and delivery-related head moulding (a, arrow).

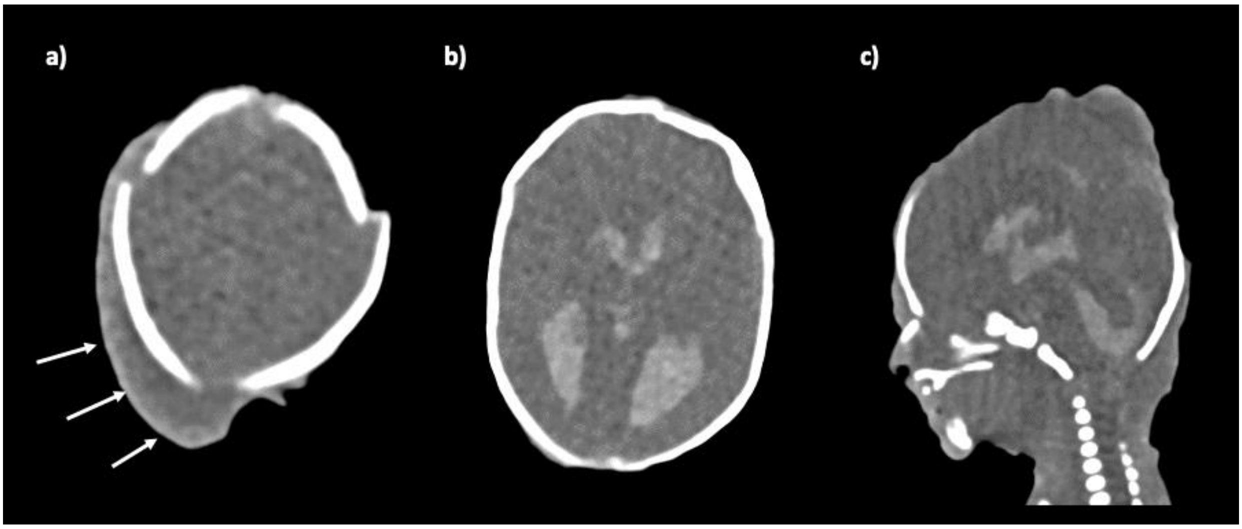

**Figure S2.** Volume rendering from an 8 year old girl who fell from height with complex skull fractures. The injuries are viewed from the patient's left side (a, c) and also looking forwards (b, d). The images on the top row (a, b) demonstrate the use of standard volume rendering, those on the bottom row (c, d) were created using cinematic rendering. The cinematic rendering technique provides a more photo-realistic appearance of the injuries and can be helpful to explain to non-medical personnel the pattern of injuries sustained during trauma.

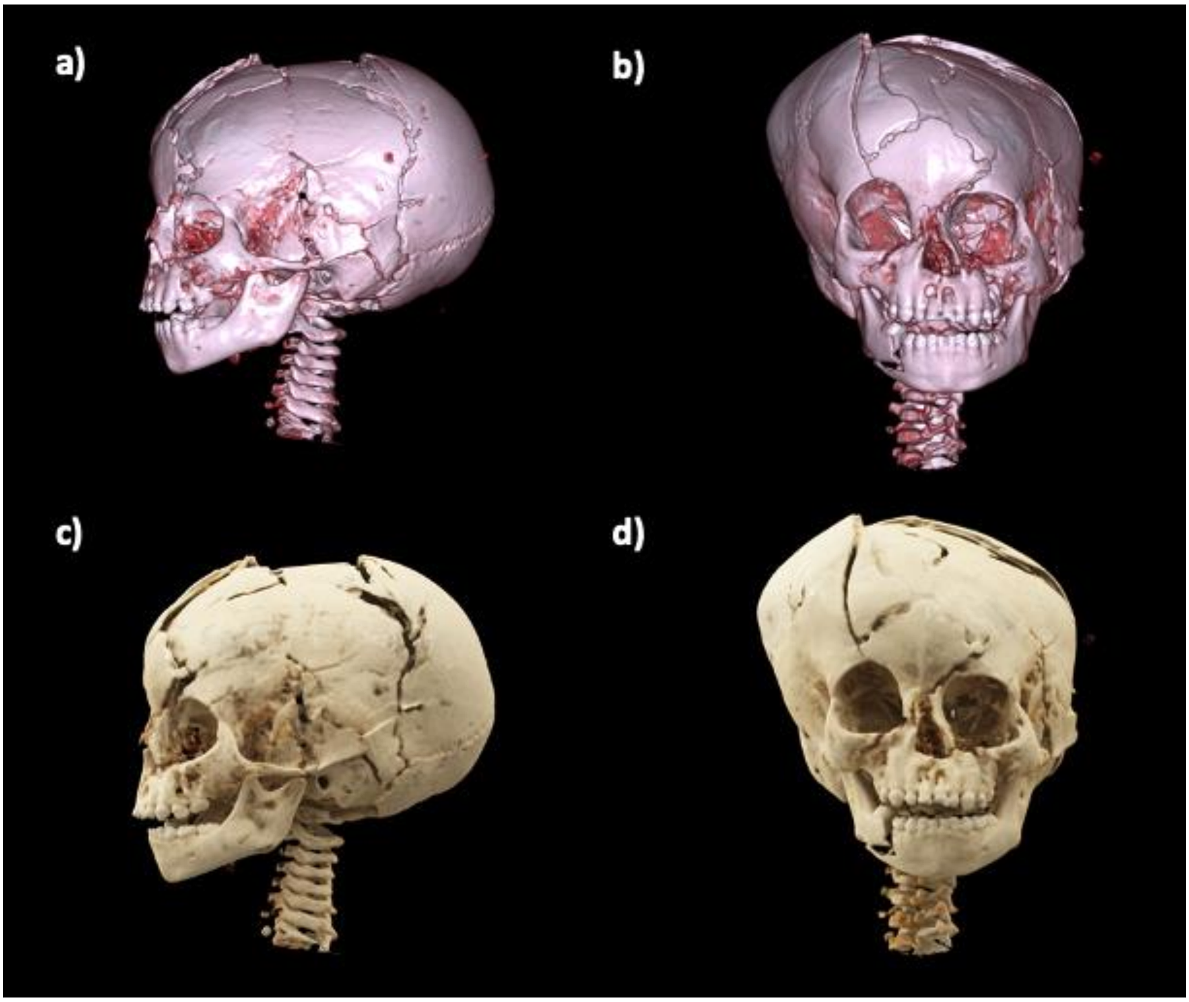

**Figure S3.** Radiographs (a, b) and axial PMCT images (c, d) showing positions of intra-osseous (IO) needle tips in a 2 year old child that were placed during attempted resuscitation. The right tibial intra-osseous needle tip is appropriately sited within the medullary cavity (a, c), but the left proximal tibial intra-osseous needle tip has traversed the bone and was misplaced (arrows, b, d).

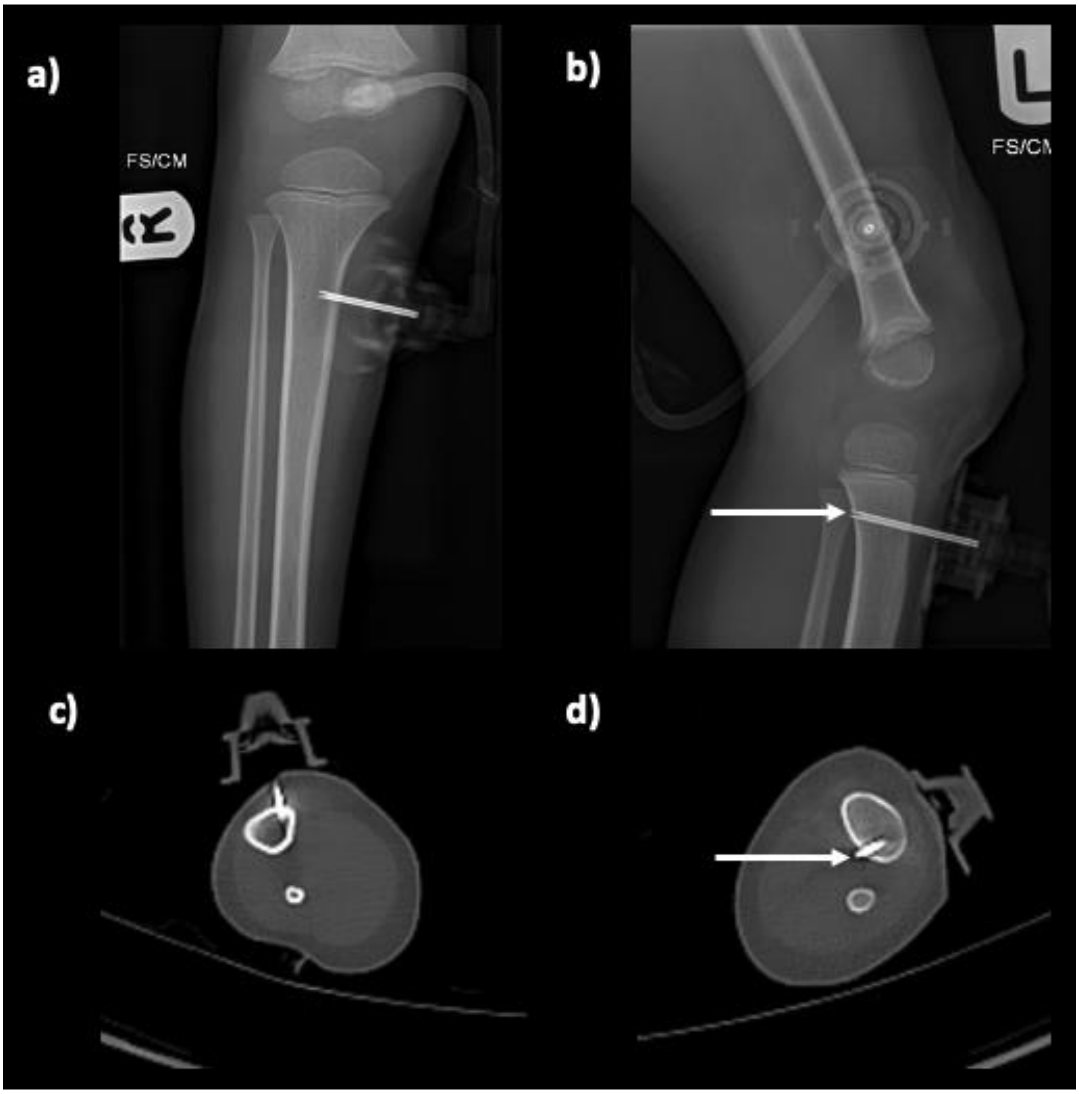

Supplement: Supplementary file 1 — ELECTRONIC SUPPLEMENTARY MATERIAL [file 13244_2024_1698_MOESM1_ESM.pdf]
